# Human nasal olfactory stem cells, purified as advanced therapy medicinal products, improve neuronal differentiation

**DOI:** 10.3389/fnins.2022.1042276

**Published:** 2022-11-17

**Authors:** Charlotte Jaloux, Maxime Bonnet, Marie Vogtensperger, Marie Witters, Julie Veran, Laurent Giraudo, Florence Sabatier, Justin Michel, Regis Legré, Gaëlle Guiraudie-Capraz, François Féron

**Affiliations:** ^1^CNRS, INP, UMR 7051, Institut de Neuropathophysiologie, Equipe Nasal Olfactory Stemness and Epigenesis (NOSE), Aix Marseille University, Marseille, France; ^2^Department of Hand Surgery and Reconstructive Surgery of the Limbs, La Timone University Hospital, Assistance Publique Hôpitaux de Marseille, Marseille, France; ^3^Faculté des Sciences du Sport de Marseille, CNRS, ISM, UMR 7287, Institut des Sciences du Mouvement Etienne-Jules MAREY, Equipe Plasticité des Systèmes Nerveux et Musculaire (PSNM), Parc Scientifique et Technologique de Luminy, Aix Marseille University, Marseille, France; ^4^Cell Therapy Department, Hôpital de la Conception, AP-HM, INSERM CIC BT 1409, Marseille, France; ^5^Aix-Marseille Université, C2VN, UMR-1263, INSERM, INRA 1260, UFR de Pharmacie, Marseille, France; ^6^Department of Otorhinolaryngology and Head and Neck Surgery, Assistance Publique des Hôpitaux de Marseille, Institut Universitaire des Systèmes Thermiques Industriels, La Conception University Hospital, Aix Marseille University, Marseille, France

**Keywords:** ecto-mesenchymal stem cell, olfactory mucosa, advanced therapy medicinal product, platelet lysate, neuronal differentiation, regeneration, olfactory stem cells as advanced therapy medicine

## Abstract

**Background:**

Olfactory ecto-mesenchymal stem cells (OE-MSC) are mesenchymal stem cells derived from the *lamina propria* of the nasal mucosa. They display neurogenic and immunomodulatory properties and were shown to induce recovery in animal models of spinal cord trauma, hearing loss, Parkinsons’s disease, amnesia, and peripheral nerve injury. As a step toward clinical practice, we sought to (i) devise a culture protocol that meets the requirements set by human health agencies and (ii) assess the efficacy of stem cells on neuron differentiation.

**Methods:**

Nasal olfactory mucosa biopsies from three donors were used to design and validate the good manufacturing process for purifying stem cells. All processes and procedures were performed by expert staff from the cell therapy laboratory of the public hospital of Marseille (AP-HM), according to aseptic handling manipulations. Premises, materials and air were kept clean at all times to avoid cross-contamination, accidents, or even fatalities. Purified stem cells were cultivated for 24 or 48 h and conditioned media were collected before being added to the culture medium of the neuroblastoma cell line Neuro2a.

**Results:**

Compared to the explant culture-based protocol, enzymatic digestion provides higher cell numbers more rapidly and is less prone to contamination. The use of platelet lysate in place of fetal calf serum is effective in promoting higher cell proliferation (the percentage of CFU-F progenitors is 15.5%), with the optimal percentage of platelet lysate being 10%. Cultured OE-MSCs do not show chromosomal rearrangement and, as expected, express the usual phenotypic markers of mesenchymal stem cells. When incorporated in standard culture medium, the conditioned medium of purified OE-MSCs promotes cell differentiation of Neuro2a neuroblastoma cells.

**Conclusion:**

We developed a safer and more efficient manufacturing process for clinical grade olfactory stem cells. With this protocol, human OE-MSCs will soon be used in a Phase I clinical based on their autologous transplantation in digital nerves with a neglected injury. However, further studies are required to unveil the underlying mechanisms of action.

## Introduction

Used in the very first clinical trial based on cell therapy, performed in the mid-1950s (Thomas et al., 1957), mesenchymal stem cells (MSC) opened a new field of therapeutic interventions. Since then, hundreds of MSC-based transplantations have been performed, in a wide range of diseases or trauma (Zhou et al., 2021). Initially restricted to bone marrow, MSCs were identified in numerous tissues, including umbilical cord, amniotic fluid, placenta, muscle, liver, skin, face [for review (Väänänen, 2005)] and many others like adipose tissue (Maria et al., 2017) and menstrual blood (Chen et al., 2019).

A subtype of MSCs, originating from the neural crest and mostly located in the face, has been isolated and categorized under the name of ecto-mesenchymal stem cells (Kaltschmidt et al., 2012). These cells, previously named mesenchymal–neural precursors, can differentiate into ectoderm and mesoderm cell types (Calloni et al., 2009) and, for clinicians willing to repair the nervous system, one specific subtype – the olfactory ecto-mesenchymal stem cell (OE-MSC) – is of particular interest. OE-MSCs belong to a nervous tissue and are involved in the permanent neurogenesis that takes place all lifelong in the olfactory mucosa (Graziadei and Graziadei, 1979). Located in the nasal cavity, this tissue is easily biopsied in vigil individuals, under local anesthesia (Girard et al., 2011).

By the end of the last century, multipotent progenitors able to give rise to neural and non-neural cells were isolated from rodent (Huard et al., 1998) and human (Roisen et al., 2001; Murrell et al., 2005) olfactory mucosa. Later on, these progenitors were characterized as a member of the ecto-mesenchymal stem cell family with neurogenic (Delorme et al., 2010) and the phenotype of OE-MSCs was extensively characterized.

This new type of stem cells, which combines some characteristics of MSCs and neural stem cells, is a good candidate for cell therapy experiments, as demonstrated in models of Parkinson’s disease (Murrell et al., 2008), deafness (Young et al., 2018) and amnesia (Nivet et al., 2011). OE-MSCs differentiate into neural cells (Murrell et al., 2008; Delorme et al., 2010; Nivet et al., 2011) exert immunomodulatory actions (Di Trapani et al., 2013) and secrete numerous factors involved in axon growth and guidance (Girard et al., 2017). We also demonstrated *in vitro* how they can cross the blood-brain barrier (Girard et al., 2017) and be transdifferentiated into dopaminergic neurons (Chabrat et al., 2019).

In anticipation of clinical applications, the time has come to design a protocol that will meet the requirements set by human health agencies. Previously, our team described methods for cultivating human OE-MSCs (Girard et al., 2011; Bonnet et al., 2020). However, the manufacture of such a medicinal product must strictly follow good manufacturing practices (GMP) from its development through to the marketing authorization. According to the European Directive No. 1394/2007, stem cell is considered as an advanced therapy medicinal product (ATMP) and as such must comply with the rules and guidelines of the European Medicines Agency. We thus devised an innovative protocol with new methods and compounds that guarantee the quality and safety of OE-MSCs.

## Materials and methods

### Study design and donor qualification

In 2019, a prospective study entitled NOSE (ClinicalTrials.gov Identifier: NCT04020367), aiming to validate the manufacture of OE-MSCs using good manufacturing practices (European Commission, 2018), was initiated. The purpose was to design a new protocol able to produce Advanced Therapeutic Medicinal Products (ATMP) for repairing peripheral nerves. Five healthy adult individuals undergoing a turbinoplasty or a septoplasty under general anesthesia were included. A complete written and oral information on the goal and procedure of this research was provided to the participants and a signed informed consent was obtained from all of them, prior to their involvement in the study. All procedures were approved by the ethical committee (2018-A00796-49) and the national competent authorities. For each individual, an olfactory mucosa biopsy and a blood sample (for genetic studies) were harvested.

Exclusion criteria were: central nervous system pathologies, pregnancy or breastfeeding women, chronic rhinosinusitis or acute rhinosinusal infection, anti-thrombotic treatment, allergy to amide-type local anesthetics or to one of their excipients, previous surgical treatment on ethmoid and/or medium turbinates, individuals with porphyria or uncontrolled epilepsia (contraindications to xylocaine and naphazoline applications), previous cervical or encephalic radiotherapy, positivity to viral serologies (VIH 1, VIH 2, VHB, VHC, HTLV1) or hemostasis anomalies (on blood works). All manufacturing processes were achieved in the LCTC (Laboratory of Culture and Cell Therapy), which is the cell therapy department of La Conception University Hospital. LCTC is approved by the health authorities (French national agency for the safety of medicines) to produce ATPMs (FR01303M for ATMP “hospital exemption,” FR 01304 M for investigational ATMP).

### Collection of olfactory mucosa biopsies

Biopsies were performed unilaterally by an Ear Nose and Throat surgeon at the level of the middle turbinate arch, using a Morscupula forceps and an endoscope, as described before (Girard et al., 2011). When the patient was deeply anesthetized and just before the planned procedure, a 2 mm^2^ biopsy was collected and transferred in a sterile tube filled with culture medium + penicillin + gentamycin (#, alpha MEM Macopharma BC0110020). The tube was then transferred to the LCTC (AP-HM, using a transport container for processing (#, ISOS 03).

### Olfactory ecto-mesenchymal stem cells

#### Olfactory ecto-mesenchymal stem cells manufacturing process

The whole culture of olfactory stem cells was performed in aseptic conditions to further therapeutic applications. After biopsy excision, all stages were performed by expert staff of the LCTC according to aseptic handling manipulations, requiring grade A laminar airflow. All consumables and raw materials were marketed for therapeutic use by suppliers.

**FIGURE 1:**
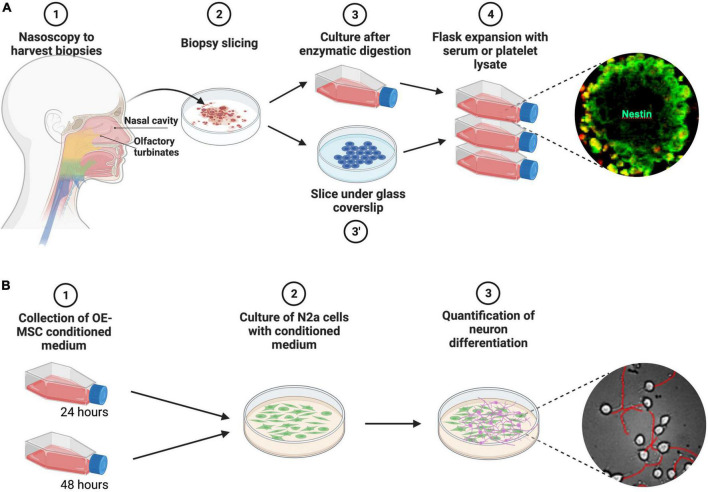
Schematic description of the two main procedures performed during the study. **(A)** Purification of olfactory stem cells. After collection, biopsies were chopped and slices were either cultivated under glass coverslips or enzymatically dissociated. Cells were then expanded with fetal calf serum or platelet lysate and characterized. **(B)** Effect of OE-MSC conditioned medium on neuron differentiation. OE-MSCs were cultivated for 24 h or 48 h and the conditioned medium was collected before being added (50/50) to the culture medium of N2a cells. To assess neuronal differentiation, the number of differentiated cells was quantified at Day 1 and Day 3.

Our previously published protocols (Girard et al., 2011, 2017; Burnouf et al., 2016) were modified in order to (1) use agreed raw materials and processing raw materials and cells bank according to ATMP good manufacturing practices (European Commission, 2018) and (2) speed up the proliferation of stem cells (Figure 1A). We compared two modes of cell individualization (enzymatic dissociation of the mucosa *versus* explant culture under coverslip, Figure 1) and two cell proliferation-inducing agent (clinical grade serum *versus* platelet lysate). Enzymatic dissociation was performed using 1 ml of collagenase NB6 (1U/ml, Nordmark Biochemicals). After a 1-h incubation at 37°C, the tissue was mechanically dissociated and the enzymatic activity was stopped by adding 9 ml of Ca-free and Mg-free PBS. The cell suspension was centrifuged at 400g for 5 min and the pellet was resuspended in a culture medium, supplemented with penicillin + gentamycin and either serum (Invitrogen) or platelet lysate (Macopharma^®^), at various concentrations. The following culture media were tested: Gibco Stem Pro (#A1014201) (medium 1), Gibco Stem Pro + CellStart (#A1014201) (medium2), DMEM/HAM F12 with 10% SVF (10094-142) with or without FGF2 (Miltenyi 130-093-838), αMEM (BC0110020) with platelet lysate. Two lines of platelet lysate (PL30 = BC0190020 vs PL100 = BC0190030), with or without gamma ray irradiation, were compared.

For comparison purpose, two similar biopsies, collected at the same location within the nasal cavity of the same donor, were cultivated in parallel, according to the two methods described above. Inter-donor reproducibility was assessed by using nasal biopsies of 2 or 3 donors for each culture. Proliferation rate was assessed by measuring the doubling time at Day 5 post-plating. All experiments were performed in triplicate or in duplicate. For each biological replicate, data were obtained by averaging measures from technical triplicates.

For the enzymatic dissociation *versus* explant culture comparison, cells were first plated in 25 cm2 flasks. When confluency was reached, cells were passaged, plated in 75 cm2 flasks and, later on, in 175 cm2 flasks. At confluency, the time of culture was assessed and the total number of cells was quantified.

#### Quality controls of cultivated olfactory ecto-mesenchymal stem cells

According to the recommendations of the European medicine agency, the quality of the ATMP were evaluated according to several criteria. The subsequent methods were used.

##### Cell number and viability

Both parameters were assessed with the LUNA-FL™ Automated Fluorescence Cell Counter. Dual fluorescence counting was achieved with Acridine Orange/Propidium Iodide Stain.

##### Cell phenotyping

Characterization of OE-MSCs was performed by flow cytometry. Aliquots of 5 × 10^5^ OE-MSCs per tube were suspended in 100 μL of PBS and stained for 20 min at room temperature in the dark with the DRAQ5 nuclear marker and pre-prepared antibodies or corresponding isotype controls in matched concentrations. The primary antibodies were anti-CD31 (IM1431U, Beckman Coulter, Brea, CA, USA), anti-CD34 (IM2709U, Beckman Coulter, Brea, CA, USA), anti-CD45 (A07785, Beckman Coulter, Brea, CA, USA), anti-CD90 (IM1839U, Beckman Coulter, Brea, CA, USA), anti-CD146 (A07483, Beckman Coulter, Brea, CA, USA), anti-S100A4 (ab41532, Abcam, Cambridge, UK) and anti-nestin (MAB5326, Sigma). They were conjugated with the following fluorochromes: fluorescein isothiocyanate (FITC), phycoerythrin (PE), phycoerythrin-Texas Red-X (ECD), and phycoerythrin-Cyanin 5.1 (PC5). Flow cytometry was performed on a NAVIOS instrument (Beckman Coulter, Brea, CA, USA) and data files were analyzed using Kaluza software (Beckman Coulter, Brea, CA, USA) with a multiparameter gating strategy.

##### Sterility and microbial assays

The following sterility and microbial assays were performed using the supernatant of each OE-MSC culture:

– Aerobic and anaerobic blood cultures, assessing the presence of germs with the BACT/ALERT^®^ 3D kit (Biomérieux).
– Turbidimetry assay determining the presence of endotoxins.
– PCR assay for *Mycoplasma sp.* detection.

##### Clonogenicity assay

The clonogenic potential of OE-MSCs was assessed using the colony formation assay with CFU-F (colony forming units-fibroblast). OE-MSCs manufactured with the irradiated PL100 were plated/seeded at a concentration of 100 cells/well in 6 well plates. The culture medium was DMEM/Ham’s F12 with 10% fetal calf serum.

Incubation was performed at 37°C under 5% CO2. The medium was renewed between Day 3 and Day 5 and then every 2–3 days, for 14 days. At the end of the culture, cells were Giemsa-stained and colonies with at least 50 cells were counted with an inversed-microscope. CFU-F percentage was determined for each well.

##### Genetic stability of olfactory ecto-mesenchymal stem cells

Olfactory ecto-mesenchymal stem cells (OE-MSCs) manufactured with the irradiated PL100 were evaluated for genetic stability using two cytogenetic assays: conventional karyotype and Array-CGH (comparative genomic hybridization).

### Neuronal differentiation assays

We performed *in vitro* experiments with the mouse neuroblastoma Neuro2a (N2a) cell line to assess the neurotrophic effects of OE-MSC-conditioned media (Figure 1B).

#### Culture of Neuro2a cell line

Neuro2a (N2a) is a neural crest-derived murine cell line that is used for studying neuronal differentiation, axonal growth and neural signaling pathways. N2a were grown and maintained in a humidified incubator in 75 cm^2^ flasks with Dulbeco’s modified eagle’s medium (DMEM)-Glutamax supplemented with 10% fetal calf serum, 1% sodium pyruvate (100 mM), 1% penicillin (100 U/ml) and streptomycin (100 μg/ml). Passaging was performed with trypsin/EDTA.

#### Neuro2a plating

N2a cells were plated in 12-well culture plates at the concentration of 20,000 cells/3.6 cm^2^ well in DMEM including glucose, 200 μM L-glutamine, 5% fetal calf serum, penicillin (100 U/ml) and streptomycin (100 μg/ml) (proliferation culture medium, named PROLIF). The medium was changed the following day to set up a new culture medium. These conditions were maintained for 4 days with a renewal of the medium on Day 2 if needed. Drug-induced differentiation was performed by adding 2 mM dibutyryl-cAMP and 1% SVF to the culture medium (differentiation culture medium named DIFF).

#### Conditioned medium-induced differentiation

OE-MSCs from 3 donors were cultured in αMEM supplemented with 5% platelet lysate and heparin (20 IU/ml). On day 4, cells were cultured in αMEM supplemented with 1% ITS (insulin, transferrin, selenium). After 24 or 48 h of culture, supernatants were collected to perform the differentiation assays. Four experimental conditions were performed in triplicate. For this purpose, N2a cells were either grown in a proliferation medium (PROLIF, negative control), or in the differentiation medium (DIFF, positive control), or in a 50/50 mixture of DIFF culture medium supplemented with OE-MSC-conditioned medium for 24 h (CM 24 h) or for 48 h (CM 48 h).

#### Neurite length assay

At Day 1 and Day 3, N2a cells were photographed with a phase contrast light microscope with a x20 magnification objective (Leica DMI4000B microscope, Leica DFC340 FX digital camera). Fifteen randomly selected fields per well were analyzed in a double-blind manner, using the Image J software and the cell counter plugin. Only cells with a neurite extending over twice the size of the diameter of the cell body were taken into consideration to determine the ratio of differentiated N2a cells *versus* the total number of cells. Neurite length was measured with the “NeuronJ” plugin of Image J software. Additional measurements (total number of extensions per field, number of extensions per cell) were performed on a random sample representative of all conditions under study.

### Statistics

All data are presented as means SEM and were analysed using GraphPad Prism6 software. Statistical analyses were performed using non-parametric one-way ANOVAs (Kruskal–Wallis) and the *post hoc* Dunn’s multiple comparison test. Differences between mean values were considered statistically significant when *p* < 0.05 (*), *p* < 0.01 (**), *p* < 0.001 (***), *p* < 0.0001 (****). Visuals were made using GraphPad Prism6, Inkscape and Adobe Photoshop software.

## Results

### A safe and highly efficient production of olfactory ecto-mesenchymal stem cells

#### Enzymatic digestion provides more rapidly a higher number of cells

**FIGURE 2:**
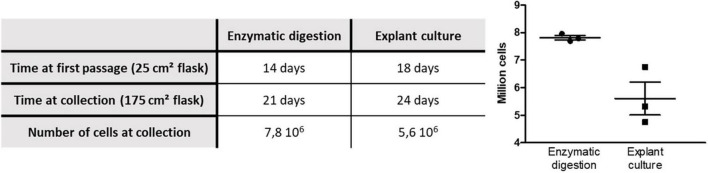
Effects of the two methods of purification on the proliferation of OE-MSCs.

As reported in Figure 2, the explant technique takes more time to reach confluency after the initial plating. The table in Figure 2 indicates that, for the explant culture, it takes 24 days to reach a total of 5.6 million cells while the enzymatic digestion technique provides nearly 8 million cells in only 21 days.

We also compared two enzymes for passaging – trypsin/EDTA *versus* TrypLE – and found no significant difference between the two cocktails: in both cases, a full dissociation was achieved, the viability was over 99% and the number of cells at Day 4 reached similar values. These findings were replicated with OE-MSCs originating from three different donors.

#### Human platelet lysate as an alternative to fetal calf serum

**FIGURE 3:**
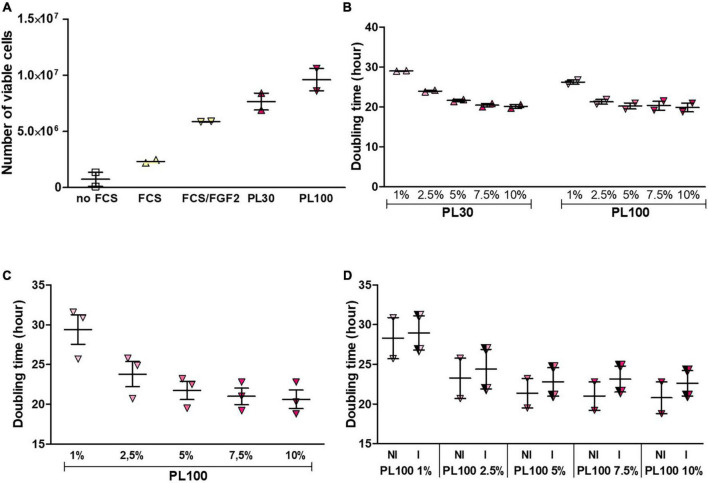
Comparison of culture conditions on cell proliferation. **(A)** Effect of platelet lysate on cell proliferation from a single donor was compared with standard medium including 10% fetal calf serum (*n* = 2). **(B)** In addition, no variability between the two tested batches – PL30 versus PL100 – is noticeable Comparison of five concentrations of platelet lysates on cell proliferation. A trend toward a reduced doubling time with increasing platelet lysate is observed (*n* = 2). **(C)** Effect of platelet lysate on cell proliferation from three donors. No statistically significant difference is observed (*n* = 3). **(D)** Effect of the sterilization procedure on cell proliferation. Gamma irradiation has no detrimental effect on doubling time (*n* = 3). I, irradiated; NI, non-irradiated.

When compared with serum, platelet lysate (v/v, 10%) induces a stronger cell proliferation: for the two platelet lysates, the fold change is 3.1 and 4.1, respectively (Figure 3A). The experiments having been carried out in duplicate, no statistical test was implemented. However, the observed trend indicates that platelet lysate is likely more efficient than serum, as far as proliferation is concerned. Increasing the percentage of platelet lysate up to 20%, is not beneficial since, in this condition, the doubling time augments (data not shown). Conversely, reducing the percentage of platelet lysate to 7.5% has no detrimental effect on cell proliferation (Figure 3B). In addition, no variability between the two tested batches – PL30 *versus* PL100 – is noticeable (Figure 3B). The experiments described above were performed with stem cells from a single donor. To identify the most efficient concentration of platelet lysate on cell proliferation, we assessed the effect of PL100 on proliferation, using stem cells from three donors. Figure 3C indicates that doubling time tends to decrease with increasing platelet lysate. However, no statistically significant difference was observed at any concentration. Gamma ray irradiation, a procedure in line with hospital health and safety guidelines, was also tested. As shown on Figure 3D, cell proliferation remains steady after sterilization of the lysate.

#### Cultivated olfactory ecto-mesenchymal stem cells are sterile, highly proliferative and their maintenance does not induce chromosomal rearrangement

Sterility and microbial assays ruled out the presence of *Mycoplasma sp.*, endotoxins, bacteria, and fungi. All final cultures were sterile. OE-MSCs display a high self-renewal activity: the mean percentage of CFU-F progenitors among the originally seeded cells was 15.5%. Putative genetic anomalies were assessed by using the techniques of karyotyping and chromosomal microarray analysis. None of these techniques detected polyploidy and/or deletions or duplications, at passage 3.

#### Purified cells express the recognized markers of olfactory ecto-mesenchymal stem cells

**FIGURE 4:**
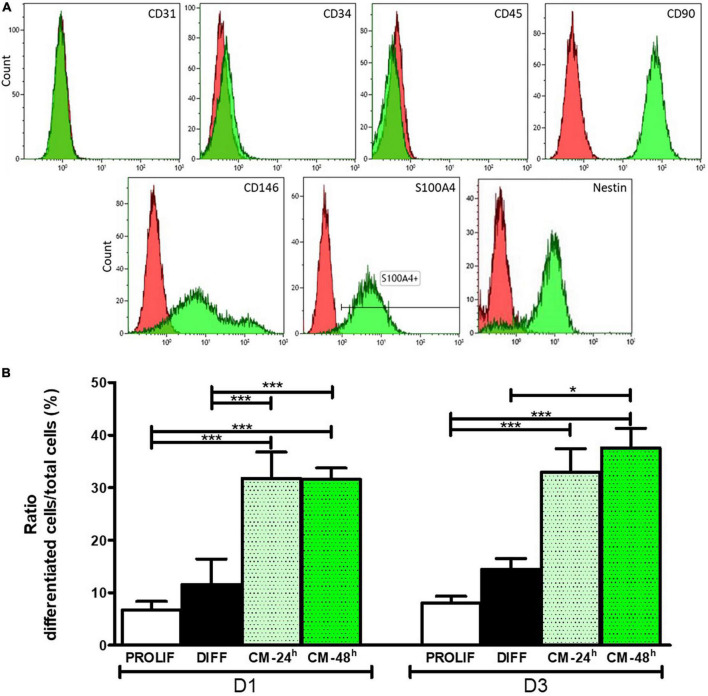
The olfactory stem cells: phenotypic characterization **(A)** and effect of OE-MSC-conditioned media on neuronal differentiation **(B)**. **(A)** Flow cytometry indicates that purified cells are negative for the usual markers of hematopoietic stem cells (CD31, CD34, CD45) and positive for the recognized markers of olfactory ecto-mesenchymal stem cells (CD90, CD146, S100A4, NESTIN). Green: cells stained with the pre-prepared antibodies; red: cells stained with isotype controls. **(B)** The percentage of differentiated N2a is significantly increased at day 1 and 3, when OE-MSC conditioned medium for 24 h or 48 h is inserted into the culture medium, except for the OE-MSC conditioned medium for 24 h when compared with the differentiation media. *p < 0.05, ***p < 0.001. PROLIF, proliferation medium; DIFF, differentiation medium; CM-24 h, OE-MSC-conditioned medium for 24 h; CM-48 h, OE-MSC-conditioned medium for 48 h.

As expected, OE-MSCs are negative for CD31, CD34, and CD45, recognized markers of hematopoietic stem cells, and positive for CD90 and CD146, surface markers of olfactory stem cells (Figure 4A). In addition, OE-MSCs express the protein S100A4, known as an intracellular marker of this specific cell type. However, to make sure that the culture was not contaminated by fibroblasts, a cell type present in the *lamina propria* that produce the proteins described above, we assessed the expression of NESTIN. It was observed that only OE-MSCs express this stem cell marker.

### Clinical grade olfactory ecto-mesenchymal stem cells-conditioned medium promotes neuron differentiation

When incubated for 3 days with a 24 h- or 48 h-OE-MSC-conditioned medium, N2a cells tend to differentiate into neurons. When compared with N2a cells cultivated with the proliferation (6.68 ± 1.65) or the differentiation (11.57 ± 4.83) medium, the ratio of differentiated cells significantly increased at day 1 with either the 24 h-OE-MSC-conditioned medium or the 48 h- OE-MSC-conditioned medium (31.75 ± 5.02 or 31.56 ± 2.17, respectively; ***p < 0.001). After 3 days of culture, the ratio of differentiated cells significantly increased for N2a cultivated with conditioned medium collected at day 1 (24 h) (32.93 ± 4.47) and day 2 (48 h) (37.56 ± 3.7), when compared with N2a in the proliferation medium (8.048 ± 1.27; ***p < 0.001) (Figure 4B). When compared with the differentiation medium (14.5 ± 2.02) at day 3, both conditioned media (24 h, 32.93 ± 4.47 and 48 h, 37.56 ± 3.7) improved N2a differentiation but the increase was statistically significant only with the 48 h conditioned medium (*p < 0.05).

## Discussion

### A safer and more efficient manufacturing process for clinical-grade olfactory stem cells

Clinical trials, based on investigational medicinal products, require manufacturing that complies with “Guidelines on Good Manufacturing Practice specific to Advanced Therapy Medicinal Products.” These guidelines are edited by the French national agency for the safety of medicines and the European commission with the regulation n°1394/2007. To fulfill these requirements, several modifications were applied. First, all cultures were performed in the cell therapy laboratory of the local public hospital (AP-HM) that got the authorization to produce ATMPs from the French national agency for the safety of medicines (EudraGMDP n°TIE/20/O/001). Recurrent visits by the agency’s managers made it possible to maintain this high level of requirement. Various steps of the culture protocol were modified to (i) suppress the use of xenogeneic products, (ii) simplify procedures and (iii) ease the fulfillment of GMP requirements. For example, the usual bovine serum was replaced by human platelet lysate, a potent surrogate for cell proliferation (Bieback, 2013). Likewise, for cell passaging, the recombinant enzyme TrypLE, efficient for dissociating other MSCs (Pinheiro de Oliveira et al., 2013; Ballesteros et al., 2020), was preferred to the usual porcine trypsin/EDTA (Murrell et al., 2008; Delorme et al., 2010) that cannot be used for clinical purposes (Hendijani, 2017). We demonstrated that both enzymes gave the same results on the proliferation and viability of olfactory stem cells.

### Enzymatic isolation is more efficient to produce more rapidly human olfactory ecto-mesenchymal stem cells

The explant technique is less time consuming and more cost effective (Matigian et al., 2010). Even more so when the enzymatic dissociation requires the addition of expensive growth factors (FGF 2, PDGF) in the culture medium. However, the purification of stem cells grown under coverslips requires additional GMP validations that, at this stage, remain uncertain. For the first time, we used a health agency-approved enzyme and, according to the post-dissociation cell count, the result was similar to those obtained with other collagenases (Murrell et al., 2005; Di Trapani et al., 2013; Batioglu-Karaaltin et al., 2016). We also compared explantation and dissociation techniques for the first time. We observed that, although enzymatic digestion was incomplete, confluency was reached faster with the latter protocol. Such a result is the consequence of a double phenomenon: some time is necessary for the cells to migrate out of the explant while the dissociated cells can spread over the whole flask.

We also compared cell viability and phenotype, according to the two techniques of purification. As opposed to other MSCs (Matigian et al., 2010), the enzymatic dissociation provides more viable OE-MSCs at day 21 (passage P2) and induces the same phenotype. In the current study as well as in a previous one (Batioglu-Karaaltin et al., 2016), purified human OE-MSCs are CD146-positive, a marker of multipotency. Intriguingly, this cell surface marker was found not expressed in the seminal study dedicated to the characterization of OE-MSCs 13.

### Platelet lysate is a powerful enhancer of olfactory ecto-mesenchymal stem cell proliferation

Bovine serum is routinely used for MSC expansion but, when cell production in clinical GMP conditions is considered, the use of animal derivatives is discouraged as it can be a source of xenogeneic antigens and zoonotic infections (European Medicines Agency, 2008; BioProcess International, 2011; European Commission, 2011). Platelet lysate is now a new standard for GMP-compliant cell manufacturing, particularly when serum-free fully defined media are not yet available for specific cell types (Oeller et al., 2021). Here we demonstrate that platelet lysate improves *in vitro* proliferation of olfactory stem cells. Previous studies report similar results for MSCs (BM-MSC, adipose derived MSC, umbilical cord MSC, dental pulp MSC, corneal stromal MSC) (Doucet et al., 2005; Schallmoser et al., 2007; Castegnaro et al., 2011; Becherucci et al., 2018; Cowper et al., 2019; Kakudo et al., 2019; Guiotto et al., 2020). In addition, it has been shown that platelet lysate maintains the differentiation potential, the immunomodulatory activity, the telomere length and chromosomal activity of the MSCs (Iudicone et al., 2014). It also supports beneficial clinical outcomes (Ballesteros et al., 2020).

Used as an effective anti-virus procedure (Viau et al., 2019), gamma irradiation does not affect the proliferation of OE-MSCs, as demonstrated here and in a previous study that assessed its role on stem cell proliferation (BM-MSC), their content in growth factors as well as their clonogenic, differentiation and immunosuppressive properties (Bense et al., 2020).

### Clinical grade olfactory ecto-mesenchymal stem cell-conditioned medium promotes neuronal differentiation

In two previous studies on rat models of peripheral nerve injury, we demonstrated the efficacy of OE-MSCs in improving motor functional recovery of the lower limb or face (Bonnet et al., 2020; Alvites et al., 2022). To assess whether the clinical grade cultured OE-MSCs stimulate axonal regrowth through extruded factors, a N2a cell line was cultivated with a 50:50 mixture of fresh differentiation medium and the conditioned medium collected from a 24 h or a 48 h-long culture of OE-MSCs. We observed that the OE-MSC-conditioned medium induces the differentiation of the N2a cells after only one day of culture. To avoid any interference of platelet lysate in the N2a differentiation process, OE-MSCs were cultivated with an ITS-supplemented medium. Within this minimalist culture medium, OE-MSCs do not proliferate or differentiate. However, further studies should assess the capacity of either insulin or transferrin or selenium (with and without addition of dbAMP) induces cell differentiation.

A previous study found similar results with non-clinical grade OE-MSC-conditioned medium. Alvites and colleagues (Bense et al., 2020) showed that OE-MSC-conditioned medium enhanced functional recovery when administered to rats with a sciatic nerve injury. They found that 48 h OE-MSC-CM as opposed to 24 h OE-MSC-CM contain more cytokines, growth factors, and neurotrophic factors beneficial for peripheral nerve regeneration. In our study we show that only 48 h OE-MSC-CM induces a statistically significant effect on neuronal differentiation of N2a. This result is probably associated to the quality of the molecules released by OE-MSCs. Alvites and colleagues reported, in the conditioned medium of OE-MSC, high concentrations of factors – e.g., MCP-1, IL-1, IL-10, VEGF – playing a central role in the regenerative process and the action of Schwann cells. To be exhaustive, we must mention another study based on the factors secreted from the olfactory cells, located in the neuroepithelium and not the lamina propria of the olfactory mucosa. It has been observed that the factors secreted by neural stem/progenitors cell (probably a cocktail of epithelial basal cells and immature neurons) promote the astrocytic differentiation of adult murine hippocampal neural precursor cells (Gómez-Virgilio et al., 2018). Among the secreted factors that seem to play a role in cell differentiation, we can cite interleukin 6, neurotrophin 4 and EGF.

The potential therapeutic benefits of OE-MSCs were also tested in animal models of various injuries/pathologies affecting the peripheral or central nervous system. Promising results have been reported in models replicating some of the symptoms/mechanisms observed in Alzheimer’s disease (Hong et al., 2022), Parkinson’s disease (Murrell et al., 2008; Farhadi et al., 2021; Simorgh et al., 2021), Huntington’s disease (Bayat et al., 2021), multiple sclerosis (Lindsay et al., 2010, 2022; Wu et al., 2013), cerebellar ataxia (Moghaddam et al., 2022) ischemic stroke (Liu et al., 2020; Zhuo et al., 2021), spinal cord injury (Lindsay et al., 2017; Stamegna et al., 2018; Voronova et al., 2020; Hamidabadi et al., 2021), amnesia (Nivet et al., 2011), hearing loss (Pandit et al., 2011). In regard to mechanisms, it has been reported that OE-MSCs secrete trophic factors, modulate the inflammatory response, phagocyte inclusions or deleterious molecules, regulate oxidative stress.

For this first experiment, we favored the use of freshly isolated conditioned medium from OE-MSC because it has been shown that storage can alter the efficacy, especially when associated with enzymatic activity (Jeyaram and Jay, 2017). Nevertheless, banking is necessary in clinical practice to provide therapeutic treatments during the acute phase of injury. This implies testing the efficacy of frozen and thawed OE-MSC-conditioned media. Furthermore, we only tested a single administration of the conditioned medium. However, nerve regeneration is a long process and repeated administrations may be beneficial.

## Conclusion

The current study reports a newly devised protocol for the purification and characterization of human OE-MSCs. The manufacturing process is in line with the rules and guidelines of national and European health agencies and the produced cells can be considered as advanced therapy medicinal products (ATMP), potentially usable in clinical trials. In addition, we demonstrate *in vitro* that OE-MSC-conditioned medium is a potent inducer of neuronal differentiation.

## Data availability statement

The raw data supporting the conclusions of this article will be made available by the authors, without undue reservation.

## Ethics statement

The studies involving human participants were reviewed and approved by Comité de Protection des Personnes, file 2018-A00796-49. The patients/participants provided their written informed consent to participate in this study.

## Author contributions

CJ, MB, MV, and MW analyzed and interpreted the data regarding the stem cells and their conditioned media. LG, FF, and GG-C performed the culture of the olfactory stem cells and the N2A cells. JV and FS helped designing the experiments. JM harvested the nasal mucosa biopsies. FF, MB, GG-C, MW, RL, and CJ had a major contributor in writing the manuscript. All authors read and approved the final manuscript.

## Abbreviations

BM-MSC: bone marrow mesenchymal stem cell
ENT: ear nose throat surgeon
FCS: fetal calf serum
LCTC: laboratory of culture and cell therapy
MSC: mesenchymal stem cell
OE-MSC: olfactory ecto-mesenchymal stem cell
PL: platelet lysate
CFU-F: colony forming units-fibroblast
CM-24 h: OE-MSC-conditioned medium for 24 h
CM-48 h: OE-MSC-conditioned medium for 48 h.
